# Performance and Energy Consumption Analysis for UWSNs with Priority Scheduling Based on Access Probability and Wakeup Threshold

**DOI:** 10.3390/s25020570

**Published:** 2025-01-19

**Authors:** Ning Li, Zhiyu Xiang, Liang Feng, Zhiqiang Gao, Jiaqi Liu, Haitao Gu

**Affiliations:** State Key Laboratory of Robotics, Shenyang Institute of Automation, Chinese Academy of Sciences, Shenyang 110016, China; lining@sia.cn (N.L.); xiangzhiyu@sia.cn (Z.X.); gaozhiqiang@sia.cn (Z.G.); liujiaqi@sia.cn (J.L.); guhaitao@sia.cn (H.G.)

**Keywords:** underwater wireless sensor networks, priority scheduling, access probability, wakeup threshold, queueing theory

## Abstract

As advancements in autonomous underwater vehicle (AUV) technology unfold, the role of underwater wireless sensor networks (UWSNs) is becoming increasingly pivotal. However, the high energy consumption in these networks can significantly reduce their operational lifespan, while latency issues can impair overall network performance. To address these challenges, a novel mixed packet forwarding strategy is developed, which incorporates a wakeup threshold and a dynamically adjusted access probability for the cluster head (CH). This approach aims to conserve energy while maintaining acceptable network latency levels. The wakeup threshold restricts the frequency of state switching for the CH, thereby reducing energy consumption. Meanwhile, the dynamic access probability regulates the influx of packets to mitigate system congestion based on current network conditions. Furthermore, to accommodate the network’s varied transmission demands, packets generated by sensor nodes (SNs) are categorized into two types according to their sensitivity to latency. A discrete−time queueing model with preemptive priority is then established to evaluate the performance of different packets and the CH. Numerical results show how different parameters affect network performance and demonstrate that the proposed mixed packet forwarding mechanism can effectively manage the trade−off between latency and energy consumption, outperforming the traditional mechanism within a specific range of parameters.

## 1. Introduction

Underwater wireless sensor networks (UWSNs) are network systems that deploy sensor nodes (SNs) in underwater environments and achieve data collection and transmission through acoustic communication, which hold broad application prospects in various fields such as ocean monitoring, resource exploration, environmental research, submarine pipeline monitoring, disaster warning, and military applications [1,2,3]. With the increasing emphasis on marine resources, the research and application of UWSNs are receiving more and more attention.

For the current research, there are mainly three structures: two−dimensional static UWSNs, three−dimensional static UWSNs, and three−dimensional UWSNs integrated with autonomous underwater vehicles (AUVs) [4]. The two−dimensional UWSN is limited to collecting data from a specific area of the seafloor. In contrast, the three−dimensional UWSN allows for the modulation of sensor node depths to gather oceanic data across various depths. The structure of the three−dimensional static UWSN is similar to that of the three−dimensional UWSNs equipped with AUVs, with the key difference being the substitution of stationary sensor nodes with mobile AUVs in the latter. This three−dimensional UWSN, enhanced by AUVs, represents an advancement over static networks, potentially boosting the communication capabilities of UWSNs. However, deploying these three−dimensional UWSN structures is challenging, and they are susceptible to damage due to the unique characteristics of underwater environments. Therefore, existing research mostly focuses on the two−dimensional static UWSN structure.

Energy consumption optimization is a core and urgent issue that needs to be addressed in UWSNs. Because SNs are typically deployed in environments that are difficult to access physically, such as deep−sea or remote water areas, the initial configuration of batteries often limits the energy supply of these nodes, and it is difficult to carry out subsequent energy replenishment or replacement [5,6,7]. Therefore, the energy consumption of SNs directly determines the lifecycle and stability of the network, and the development of energy−saving technologies is particularly crucial. Cluster structure is the most efficient energy−saving method in UWSNs, whose core principle is to organize a large number of SNs into clusters [8]. Each cluster is led by a cluster head (CH), responsible for coordinating and managing the data collection, processing, and transmission of nodes within the cluster [9].

In actual underwater network environments, data sensitivity to transmission latency often varies [10]. Sudden events in environmental monitoring (such as ocean pollution spills, early warning of natural disasters, etc.) usually require more rapid forwarding. In contrast, information with lower time sensitivity (such as long−term climate trend analysis and monitoring of periodic changes in ecosystems) can tolerate a certain degree of latency. Therefore, designing a reasonable data classification mechanism can more intelligently allocate limited resources and improve the network’s overall performance.

Congestion control is particularly important and regarded as one of the most significant challenges [11,12]. Congestion not only reduces network throughput but may also lead to packet loss, affecting the reliability and stability of the network. Frequent retransmissions consume valuable energy from nodes, accelerating their energy depletion and shortening the network’s lifespan. Therefore, it is necessary to introduce reasonable congestion control mechanisms, dynamically adjust data transmission, balance network loads, and avoid or reduce congestion.

This paper proposes a mixed packet forwarding mechanism with a priority schedule based on dynamic access probability and wakeup threshold. The wakeup threshold controls the system’s state switching and saves system energy consumption. The dynamic access probability controls packet access based on the system’s implementation status, reducing system congestion. The following are the primary contributions of this paper.

1. A mixed packet forwarding mechanism is suggested to satisfy the diversity needs of data transmission while balancing average latency and network energy consumption.
2. A three−dimensional Markov−chain (3DMC) model with preemptive priority is established to evaluate the performance of the proposed mechanism.
3. Numerical results show how various parameters affect system performance and how effective the proposed method is.

The remainder of the paper is structured as below. In Section 2, we review the relevant literature. We provide a mechanism description and modeling analysis of the forwarding mechanism with priority scheduling based on dynamic access probability and wakeup threshold in Section 3. The performance expressions for two different priority packets and the CH are given in Section 4. In Section 5, we analyze how various parameters affect system performance through numerical results and evaluate the effectiveness of the proposed mechanism in balancing average latency and energy consumption. In Section 6, we summarize our work. The complementary probability event is expressed using the overbar mark, and Table 1 displays the symbols used in this paper along with their meanings.

## 2. Related Works

In UWSNs, queueing theory is widely used to analyze network performance and optimize network topology [13,14,15]. In [13], a data acquisition method was proposed based on the queueing model and the genetic algorithm for underwater acoustic collaborative sensor networks. This method modeled the process of each SN sending packets to the CH as a single service station hybrid M/M/1/K queueing model. The results demonstrated that this method could effectively reduce packet loss rate. In [14], a latency−sensitive underwater wireless optical network was analyzed. An M/G/1 queueing model was constructed to quantify the system performance, and the end−to−end latency and blocking rate were evaluated in different scenarios through the numerical results. In [15], a number of charging strategies were suggested for three−dimensional charging UWSNs. An M/G/1 queueing model was used to simulate the energy mule charging process. The proposed scheme was verified through simulation experiments to be energy−saving and time−saving and to guarantee the efficient use of resources. The above literature adopted the continuous−time queueing theory for modeling. However, we observe that the digital features of contemporary network communication are better suited to the discrete−time queueing theory [16].

Sleep/wakeup mode is an effective energy−saving strategy to improve network lifetime. Due to the limited energy of underwater SNs, the network’s lifespan can be extended significantly by reasonably arranging the wakeup and sleep states of nodes. In [17], an adaptive sleep/wakeup scheduling method was proposed. The timeline was divided into multiple time slots, and each node was allowed to autonomously decide whether to sleep, listen, or send in one−time slot. In [18], a three−dimensional space−moving target tracking method based on the Kalman filter was proposed, and the sleep/wakeup strategy was used to save the system’s energy consumption. In this method, only part of the nodes closer to the target path would be woken up and participate in the tracking. The simulation results demonstrated that the average energy consumption could be reduced effectively. These studies designed different sleep/wake modes for application scenarios to balance energy consumption and performance, but they did not limit the switching between the two states. We note that switching between the sleep and wakeup states also takes a lot of energy and time, so this paper introduces a wakeup threshold to reduce the frequency of state switching [19]. In the wakeup threshold mechanism, wakeup is performed after a certain amount of information has been accumulated, which will also increase the average latency to a certain extent. Therefore, it is necessary to introduce a reasonable access control mechanism to mitigate this adverse effect.

The tail−drop method is the default congestion management technique for wireless networks, which adopts the principle of first−come−first−serve. When the cache is full, newly arrived packets will be dropped directly [20]. This method can only react when congestion occurs in the system, and cannot prevent congestion from occurring. Random early detection (RED) is a widely used method of queue management that can effectively prevent system congestion [21,22]. In [23], a variety of queueing models were established and analyzed according to whether there were RED and tail−drop mechanisms or priority packets. Numerical results showed that dividing packets into multiple priorities could improve the quality of service of the network, and the introduction of RED and tail−drop mechanisms could effectively alleviate congestion. However, this RED method required two thresholds (minimum and maximum thresholds) and a maximum probability for joint regulation, which is more complex to implement. In [24], considering the ignorance of queue information, a probabilistic access method for wireless sensor networks was proposed to control system congestion. When a sensor was in the sleep state, it received newly arrived packets with probability *p*; when it was in the wakeup state, it received the newly arrived packets with probability *q*. Based on this strategy, a Markov−chain model was constructed, and numerical results verified its effectiveness. However, this method of using a fixed value as the access probability was difficult to adapt to the changing network environment. In [25], an access control strategy with a dynamic access threshold was proposed. A reasonable dynamic access threshold was set according to different packet arrival rates to constrain the access behavior of packets. Numerical experiments verified the effectiveness of the strategy in energy saving. This method was similar to the wakeup threshold used in this paper; however, if congestion is to be effectively avoided, other mechanisms need to be introduced to regulate it. Therefore, we propose a dynamic access probability mechanism that will reduce access probability when many packets are retained in the system based on the influence of real−time queue length rather than the packet arrival rate. Compared with the RED method used in literature [23], the dynamic access probability mechanism used in this paper requires fewer control parameters and is simpler to implement. Compared with the fixed access probability method in [24], the dynamic access probability mechanism is more suitable for dynamically changing network environments. Compared with the method in [25], our method can alleviate congestion further.

In summary, this paper studies the packet forwarding mechanism of the CH in UWSNs with different latency sensitivities. Taking into account the trade−off between latency and energy consumption, wakeup threshold and dynamic access probability are introduced to control the CH’s state switching and packet access behavior, respectively. By constructing a discrete−time queueing model, we provide a performance analysis and energy consumption evaluation method to effectively balance energy consumption and latency. The novelty of this paper is summarized as follows:

1. A packet grading mechanism with preemptive priority is proposed to satisfy the diversified latency tolerance of packets.
2. A hybrid packet forwarding mechanism is proposed to effectively balance latency and energy consumption.
3. A new performance quantitative analysis method using discrete−time queueing theory is provided.

## 3. System Model

### 3.1. Mechanism Description

**Figure 1:**
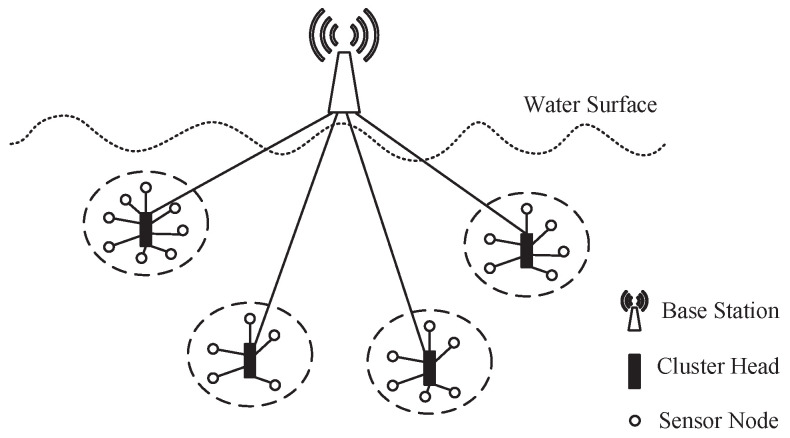
The UWSN architecture we considered in this paper [26].

Figure 1 shows the architecture of the UWSN considered in this paper.

As shown in Figure 1, we consider a homogeneous UWSN consisting of three types of nodes: the base station (BS), SNs, and CHs. Each SN or CH has the same physical characteristics and operating mode. SNs are cheaper and have less energy than CHs, which cannot directly communicate with the BS. CHs have more energy and gather packets sent by SNs and forward them to the BS.

SNs can generate two types of packets based on different perceived environmental content: emergency packets and non−emergency packets. The information carried by emergency packets is highly urgent and requires timely transmission to the BS. Non−emergency packets have lower real−time transmission requirements and can tolerate significant queueing delays. Therefore, emergency packets are given higher priority and can preempt non−emergency packets’ cluster head forwarding rights. Considering that the packets generated by each SN arrive at each CH without discrimination, we mainly focus on analyzing a single CH system to evaluate the overall performance of this architecture.

**Figure 2:**
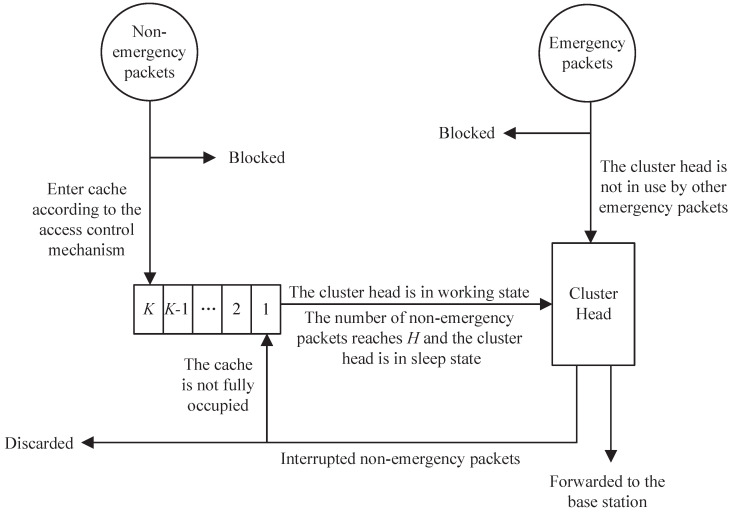
The packet forwarding mechanism.

Figure 2 describes the packet forwarding mechanism in the system.

In Figure 2, considering the high sensitivity of emergency packets to latency and the high throughput requirements of non−emergency packets, a cache with capacity K(K≥0) is set for only non−emergency packets. However, suppose many non−emergency packets are stuck in the system. In that case, it may reduce the forwarding performance and make the waiting packets lose their timeliness. Therefore, an access control mechanism is introduced to determine whether non−emergency packets can enter the cache. Non−emergency packets will dynamically change their access probability $f(\bar{f}=1-f)$ based on the amount of packets. The more packets there are, the less likely the incoming non−emergency packets can access the system. The access probability *f* is expressed as follows.

(1) $$f=1-\frac{N(1-f_{\min})}{K+1}$$

where *N* is the amount of packets, $f_{min}\left(0\leq f_{min}\leq 1\right)$ is the minimum value of *f*, and *K* is the cache size. Due to the fact that the cache can hold up to *K* packets and the CH can hold one packet, *N* will not exceed K+1.

For the sake of simplicity, let $\alpha =\frac{K+1}{1-f_{\min}},\alpha \in \left[K+1,\infty \right)$ and refer to α as the adjustment factor of *f*. Therefore, the access probability *f* can be succinctly expressed as follows.

(2) $$f=1-\frac{N}{\alpha }$$

**Figure 3:**
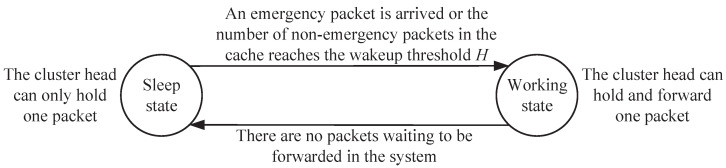
The state switching mechanism of the CH.

We define the packet forwarding process by the CH as the working state; otherwise, it is defined as the sleep state. When the CH is in sleep state, it can be regarded as a cache space that can only hold one packet rather than forward it. Considering that the transition of the CH from the sleep state to working state requires a significant amount of energy consumption, a wakeup control mechanism controlled by a wakeup threshold H(0≤H≤K) is introduced to reduce the state switching frequency of the CH. The setting of *H* cannot exceed the cache size *K*, so that the system can accumulate *H* non−emergency packets before being awakened, thereby reducing the frequency of state switching. Only when the CH is in the working state or the amount of non−emergency packets in the cache reaches *H* will the CH forward the non−emergency packets in the cache. On the contrary, once emergency packets arrive at the CH, the CH will forward them directly. Based on the wakeup control mechanism, the state swtching process of the CH can be described in Figure 3.

**Figure 4:**
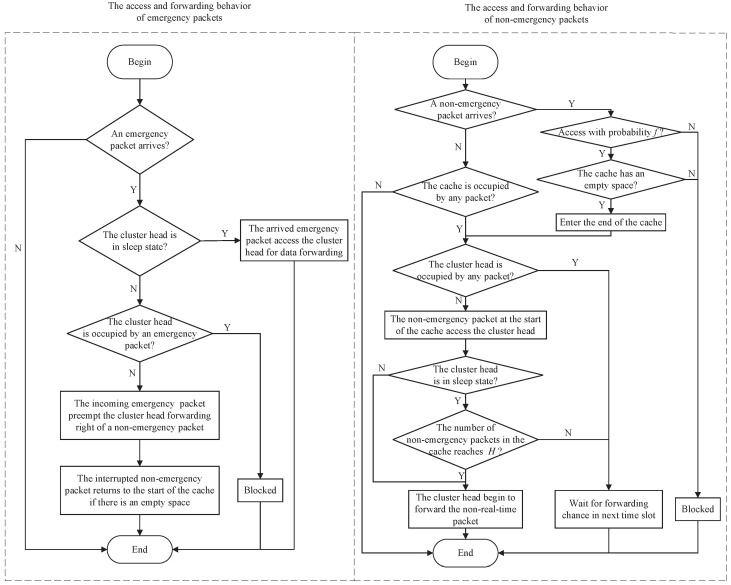
The access and forwarding behavior of two kinds of packets in each time slot.

Combining Figure 2 and Figure 3, we can summarize the access and forwarding behavior of two kinds of packets in each time slot as Figure 4.

As shown in Figure 4, when there is an incoming emergency packet, if the CH is in the sleep state at this time, this emergency packet will directly access the CH for data forwarding and the CH will enter working state (if the CH stores a non-emergency packet at this time, this non-emergency will be preempted). If the CH is in the working state and the forwarded packet is a non−emergency packet at this time, this incoming emergency packet will preempt the forwarding priority of the non−emergency packet, and the interrupted non−emergency packet returns to the start of the cache to wait for further forwarding when there is a space in the cache; if the CH is in the working state at this time and the forwarded packet is an emergency packet, the incoming emergency packet will be blocked and discarded. To sum up, as long as there is an emergency packet in the system, the CH will enter the working state.

When there is an incoming non−emergency packet in the system, this non−emergency packet will access the end of the cache with access probability *f* to wait for data forwarding if there is a space in the cache. Otherwise, it will be discarded. Moreover, if other packets do not occupy the CH, the non−emergency packet at the start of the cache will access the CH (if the CH is still in sleep mode at this time, it only temporarily stores the non−emergency packet without forwarding; if the CH is in working state, the non−emergency packet in the CH will be forwarded directly). If the CH is in the sleep state and the amount *N* of packets in the cache reaches *H*, the CH will switch to the working state and begin to forward this packet; otherwise, the non−emergency packets in the system will wait for next forwarding chance. To sum up, the access probability *f* only controls the possibility of non−emergency packets accessing the system. Whether the CH can switch from the sleep state to the working state depends on whether the number *N* of non−emergency packets in the system exceeds the wakeup threshold *H*.

### 3.2. Model Building

In UWSNs, random events (such as temperature change, humidity change, object movement, etc.) perceived by SNs occur randomly. The occurrence of these events can be regarded as a random process, and its probability distribution can be used to describe the frequency of occurrence of events. When these events are perceived by the SNs and packets are generated, the arrival of packets is also random. Therefore, we assume that the arrival process of packets is a Bernoulli process. Specifically, the Bernoulli process is a simple stochastic process in which the occurrence or absence of events within each time interval is independent, and the probability of events occurring within each time interval is constant. In this way, we can more accurately analyze and optimize the performance of UWSNs, ensuring that the network can efficiently process and transmit the perceived event information.

We divide the time axis into infinite time slots with the same interval, expressed as t=1,2,3,⋯. We assume that arrival intervals of emergency and non−emergency packets follow the geometric distribution, and the arrival rates are $p_{1}\left(0<p_{1}<1,{\bar{p}}_{1}=1-p_{1}\right)$ and $p_{2}\left(0<p_{2}<1,{\bar{p}}_{2}=1-p_{2}\right)$, respectively. The forwarding time of the CH for emergency and non−emergency packets follows geometric distribution, and the service rates are $s_{1}\left(0<s_{1}<1,{\bar{s}}_{1}=1-s_{1}\right)$ and $s_{2}\left(0<s_{2}<1,{\bar{s}}_{2}=1-s_{2}\right)$. Based on the regulation of the Early Arrival System, the packet arrival event and departure occur at the start $\left(t,t^{+}\right)$ and end $\left(t^{-},t\right)$ of each time slot, respectively [27]. Let $R_{t}=i\left(i=0,1,2,\ldots ,K+1\right)$, $S_{t}=j\left(j=0,1\right)$ and $T_{t}=k\left(k=0,1\right)$, where *i*, *j*, and *k* are three state−variables, $R_{t}$ represents the amount of total packets in the system at time $t^{+}$, $S_{t}$ represents the amount of emergency packets in the system at time $t^{+}$, and $T_{t}$ represents the state of the CH at time $t^{+}$. When $T_{t}=0$, it indicates that the CH is in the sleep state; otherwise, when $T_{t}=1$, it indicates that the CH is in the working state. Then $\left\{R_{t},S_{t},T_{t}\right\}$ constitutes a 3DMC with state space Ω, and Ω is expressed as follows.

(3) $$\Omega =\left\{\begin{array}{cc} \left\{(0,0,0)\cup \{(i,0,1)\cup (i,1,1):1\leq i\leq K+1\}\right\} & H=0\\ \left\{\{(i,0,0):0\leq i\leq H\}\cup \{(i,0,1)\cup (i,1,1)\}:1\leq i\leq K+1\right\} & 1\leq H\leq K \end{array}\right.$$

According to the structure of the state space Ω, we can obtain that there are (2K+H+3) states in the system. We mark W as the one−step transition probability matrix of the 3DMC, and W can be expressed by block matrices as follows based on the change of the amount of total packets in the one−step transition.

(4) $$W={\left(\begin{array}{ccccccc} W_{0,0} & W_{0,1} & W_{0,2} & & & 0 & \\ W_{1,0} & W_{1,1} & W_{1,2} & W_{1,3} & & & \\ & W_{2,1} & W_{2,2} & W_{2,3} & W_{2,4} & & \\ & & \ddots & \ddots & \ddots & \ddots & \\ & & & W_{K-1,K-2} & W_{K-1,K-1} & W_{K-1,K} & W_{K-1,K+1}\\ & 0 & & & W_{K,K-1} & W_{K,K} & W_{K,K+1}\\ & & & & & W_{K+1,K} & W_{K+1,K+1} \end{array}\right)}_{\left(2K+H+3)\times (2K+H+3\right)}$$

We will give each block matrix $W_{m,n}$ in detail, where *m* and *n* represent the amount of total packets before and after the one−step transition.

1. When m=0, it indicates that the amount of packets is zero before the one−step transition, and the transition submatrices are given as follows.
  (5) $$W_{0,0}={\left({\bar{p}}_{1}{\bar{p}}_{2}\right)}_{1\times 1}$$
  (6) $$W_{0,1}=\left\{\begin{array}{cc} {\left(\begin{array}{cc} {\bar{p}}_{1}p_{2} & p_{1}{\bar{p}}_{2} \end{array}\right)}_{1\times 2} & H=0\\ {\left(\begin{array}{ccc} {\bar{p}}_{1}p_{2} & 0 & p_{1}{\bar{p}}_{2} \end{array}\right)}_{1\times 3} & 1\leq H\leq K \end{array}\right.$$
  (7) $$W_{0,2}=\left\{\begin{array}{cc} {\left(\begin{array}{cc} 0 & p_{1}p_{2} \end{array}\right)}_{1\times 2} & H=0\\ {\left(\begin{array}{ccc} 0 & 0 & p_{1}p_{2} \end{array}\right)}_{1\times 3} & 1\leq H\leq K \end{array}\right.$$
2. When m=1 and n=0, due to the special dimension of the submatrix, its form is given as follows.
  (8) $$W_{1,0}=\left\{\begin{array}{cc} {\left(\begin{array}{cc} s_{2}{\bar{p}}_{1}\left({\bar{p}}_{2}+p_{2}\frac{1}{\alpha }\right) & s_{1}{\bar{p}}_{1}\left({\bar{p}}_{2}+p_{2}\frac{1}{\alpha }\right) \end{array}\right)}_{2\times 1}^{T} & H=0\\ {\left(\begin{array}{ccc} 0 & s_{2}{\bar{p}}_{1}\left({\bar{p}}_{2}+p_{2}\frac{1}{\alpha }\right) & s_{1}{\bar{p}}_{1}\left({\bar{p}}_{2}+p_{2}\frac{1}{\alpha }\right) \end{array}\right)}_{3\times 1}^{T} & 1\leq H\leq K \end{array}\right.$$
3. When 1≤m≤K and n=m−1(n≠0), $W_{m,n}$ is expressed as follows.
  (9) $$W_{m,n}=\left\{\begin{array}{cc} {\left(\begin{array}{ccc} 0 & 0 & 0\\ 0 & s_{2}{\bar{p}}_{1}\left({\bar{p}}_{2}+p_{2}\frac{m}{\alpha }\right) & 0\\ 0 & s_{1}{\bar{p}}_{1}\left({\bar{p}}_{2}+p_{2}\frac{m}{\alpha }\right) & 0 \end{array}\right)}_{3\times 3} & m\leq H\;\\ {\left(\begin{array}{ccc} 0 & s_{2}{\bar{p}}_{1}\left({\bar{p}}_{2}+p_{2}\frac{m}{\alpha }\right) & 0\\ 0 & s_{1}{\bar{p}}_{1}\left({\bar{p}}_{2}+p_{2}\frac{m}{\alpha }\right) & 0 \end{array}\right)}_{2\times 3} & m=H+1\;\\ {\left(\begin{array}{cc} s_{2}{\bar{p}}_{1}\left({\bar{p}}_{2}+p_{2}\frac{m}{\alpha }\right) & 0\\ s_{1}{\bar{p}}_{1}\left({\bar{p}}_{2}+p_{2}\frac{m}{\alpha }\right) & 0 \end{array}\right)}_{2\times 2} & \;m\geq H+2 \end{array}\right.$$
4. When 1≤m≤K and n=m, $W_{m,n}$ is expressed as follows.
  (10) $$\begin{array}{c} W_{m,n}=\left\{\begin{array}{cc} {\left(\begin{array}{ccc} {\bar{p}}_{1}\left({\bar{p}}_{2}+p_{2}\frac{m}{\alpha }\right) & 0 & 0\\ 0 & s_{2}{\bar{p}}_{1}p_{2}\left(1-\frac{m}{\alpha }\right)+{\bar{s}}_{2}{\bar{p}}_{1}\left({\bar{p}}_{2}+p_{2}\frac{m}{\alpha }\right) & s_{2}p_{1}\left({\bar{p}}_{2}+p_{2}\frac{m}{\alpha }\right)\\ 0 & s_{1}{\bar{p}}_{1}p_{2}\left(1-\frac{m}{\alpha }\right) & \left(s_{1}p_{1}+{\bar{s}}_{1}\right)\left({\bar{p}}_{2}+p_{2}\frac{m}{\alpha }\right) \end{array}\right)}_{3\times 3} & m\leq H\\ {\left(\begin{array}{cc} s_{2}{\bar{p}}_{1}p_{2}\left(1-\frac{m}{\alpha }\right)+{\bar{s}}_{2}{\bar{p}}_{1}\left({\bar{p}}_{2}+p_{2}\frac{m}{\alpha }\right) & s_{2}p_{1}\left({\bar{p}}_{2}+p_{2}\frac{m}{\alpha }\right)\\ s_{1}{\bar{p}}_{1}p_{2}\left(1-\frac{m}{\alpha }\right) & \left(s_{1}p_{1}+{\bar{s}}_{1}\right)\left({\bar{p}}_{2}+p_{2}\frac{m}{\alpha }\right) \end{array}\right)}_{2\times 2} & m\geq H+1 \end{array}\right. \end{array}$$
5. When 1≤m≤K−1 and n=m+1, $W_{m,n}$ is expressed as follows.
  (11) $$\begin{array}{c} W_{m,n}=\left\{\begin{array}{cc} {\left(\begin{array}{ccc} {\bar{p}}_{1}p_{2}\left(1-\frac{m}{\alpha }\right) & 0 & p_{1}\left({\bar{p}}_{2}+p_{2}\frac{m}{\alpha }\right)\\ 0 & {\bar{s}}_{2}{\bar{p}}_{1}p_{2}\left(1-\frac{m}{\alpha }\right) & {\bar{s}}_{2}p_{1}\left({\bar{p}}_{2}+p_{2}\frac{m}{\alpha }\right)+s_{2}p_{1}p_{2}\left(1-\frac{m}{\alpha }\right)\\ 0 & 0 & \left(s_{1}p_{1}+{\bar{s}}_{1}\right)p_{2}\left(1-\frac{m}{\alpha }\right) \end{array}\right)}_{3\times 3} & \;m\leq H-1\\ {\left(\begin{array}{cc} {\bar{p}}_{1}p_{2}\left(1-\frac{m}{\alpha }\right) & p_{1}\left({\bar{p}}_{2}+p_{2}\frac{m}{\alpha }\right)\\ {\bar{s}}_{2}{\bar{p}}_{1}p_{2}\left(1-\frac{m}{\alpha }\right) & {\bar{s}}_{2}p_{1}\left({\bar{p}}_{2}+p_{2}\frac{m}{\alpha }\right)+s_{2}p_{1}p_{2}\left(1-\frac{m}{\alpha }\right)\\ 0 & \left(s_{1}p_{1}+{\bar{s}}_{1}\right)p_{2}\left(1-\frac{m}{\alpha }\right) \end{array}\right)}_{3\times 2} & m=H\\ {\left(\begin{array}{cc} {\bar{s}}_{2}{\bar{p}}_{1}p_{2}\left(1-\frac{m}{\alpha }\right) & {\bar{s}}_{2}p_{1}\left({\bar{p}}_{2}+p_{2}\frac{m}{\alpha }\right)+s_{2}p_{1}p_{2}\left(1-\frac{m}{\alpha }\right)\\ 0 & \left(s_{1}p_{1}+{\bar{s}}_{1}\right)p_{2}\left(1-\frac{m}{\alpha }\right) \end{array}\right)}_{2\times 2} & m\geq H+1 \end{array}\right. \end{array}$$
6. When m=K and n=m+1, $W_{m,n}$ is expressed as follows.
  (12) $$W_{m,n}=\left\{\begin{array}{cc} {\left(\begin{array}{cc} {\bar{p}}_{1}p_{2}\left(1-\frac{K}{\alpha }\right) & p_{1}\\ {\bar{s}}_{2}{\bar{p}}_{1}p_{2}\left(1-\frac{K}{\alpha }\right) & {\bar{s}}_{2}p_{1}+s_{2}p_{1}p_{2}\left(1-\frac{K}{\alpha }\right)\\ 0 & \left(s_{1}p_{1}+{\bar{s}}_{1}\right)p_{2}\left(1-\frac{K}{\alpha }\right) \end{array}\right)}_{3\times 2} & m=H\;\\ {\left(\begin{array}{cc} {\bar{s}}_{2}{\bar{p}}_{1}p_{2}\left(1-\frac{K}{\alpha }\right) & {\bar{s}}_{2}p_{1}+s_{2}p_{1}p_{2}\left(1-\frac{K}{\alpha }\right)\\ 0 & \left(s_{1}p_{1}+{\bar{s}}_{1}\right)p_{2}\left(1-\frac{K}{\alpha }\right) \end{array}\right)}_{2\times 2} & m\geq H+1\; \end{array}\right.$$
7. When 1≤m≤K−1 and n=m+2, $W_{m,n}$ is expressed as follows.
  (13) $$W_{m,n}=\left\{\begin{array}{cc} {\left(\begin{array}{ccc} 0 & 0 & p_{1}p_{2}\left(1-\frac{m}{\alpha }\right)\\ 0 & 0 & {\bar{s}}_{2}p_{1}p_{2}\left(1-\frac{m}{\alpha }\right)\\ 0 & 0 & 0 \end{array}\right)}_{3\times 3} & \;m\leq H-2\\ {\left(\begin{array}{cc} 0 & p_{1}p_{2}\left(1-\frac{m}{\alpha }\right)\\ 0 & {\bar{s}}_{2}p_{1}p_{2}\left(1-\frac{m}{\alpha }\right)\\ 0 & 0 \end{array}\right)}_{3\times 2} & H-1\leq m\leq H\;\\ {\left(\begin{array}{cc} 0 & {\bar{s}}_{2}p_{1}p_{2}\left(1-\frac{m}{\alpha }\right)\\ 0 & 0 \end{array}\right)}_{2\times 2} & m\geq H+1\; \end{array}\right.$$
8. When m=K+1 and n=m−1(n≠0), $W_{m,n}$ is expressed as follows.
  (14) $$W_{m,n}=\left\{\begin{array}{cc} {\left(\begin{array}{ccc} 0 & s_{2}{\bar{p}}_{1}\left({\bar{p}}_{2}+p_{2}\frac{K+1}{\alpha }\right) & 0\\ 0 & s_{1}{\bar{p}}_{1}\left({\bar{p}}_{2}+p_{2}\frac{K+1}{\alpha }\right) & 0 \end{array}\right)}_{2\times 3} & m=H+1\;\\ {\left(\begin{array}{cc} s_{2}{\bar{p}}_{1}\left({\bar{p}}_{2}+p_{2}\frac{K+1}{\alpha }\right) & 0\\ s_{1}{\bar{p}}_{1}\left({\bar{p}}_{2}+p_{2}\frac{K+1}{\alpha }\right) & 0 \end{array}\right)}_{2\times 2} & m\geq H+2\; \end{array}\right.$$
9. When m=n=K+1, $W_{m,n}$ is expressed as follows.
  (15) $$W_{m,n}={\left(\begin{array}{cc} s_{2}{\bar{p}}_{1}p_{2}\left(1-\frac{k+1}{\alpha }\right)+{\bar{s}}_{2}{\bar{p}}_{1} & p_{1}\\ s_{1}{\bar{p}}_{1}p_{2}\left(1-\frac{k+1}{\alpha }\right) & s_{1}p_{1}+{\bar{s}}_{1} \end{array}\right)}_{2\times 2}$$

All submatrices have been discussed, and the transition probability matrix W can be further obtained. We define $\pi_{x,y,z}$ as the steady−state probability distribution of the 3DMC, and $\pi_{x,y,z}$ is expressed as follows.

(16) $$\pi_{x,y,z}=\lim_{t\to \infty }P\left\{R_{t}=x;S_{t}=y;T_{t}=z:0\leq x\leq K+1,0\leq y\leq 1,0\leq z\leq 1\right\}$$

We can further define the steady−state vector Π as follows.

(17) $$\Pi =\left\{\begin{array}{cc} \left(\pi_{000},\pi_{1,0,1},\pi_{1,1,1},\ldots ,\pi_{K+1,0,1},\pi_{K+1,1,1}\right) & H=0\\ (\pi_{000},\pi_{1,0,0},\pi_{1,0,1},\pi_{1,1,1},\ldots ,\pi_{H,0,0},\pi_{H,0,1},\pi_{H,1,1},\\ \pi_{H+1,0,1},\pi_{H+1,1,1},\ldots ,\pi_{K+1,0,1},\pi_{K+1,1,1}) & 1\leq H\leq K \end{array}\right.$$

According to the structure of the matrix W, the 3DMC is irreducible, aperiodic, and normally recurrent. Based on the equilibrium equation and normalization condition [28], we can obtain the following equations.

(18) $$\left\{\begin{array}{c} \Pi W=\Pi \\ \Pi e=1 \end{array}\right.$$

where e is a column vector with all 1 s. A numerical calculation method [29] for calculating the equations above is provided in Table 2.

## 4. Performance Metrics

### 4.1. Performance Metrics for Emergency Packets

#### 4.1.1. Emergency Packets’ Blocking Rate

When an emergency packet arrives, if other emergency packets occupy the CH at this time, the incoming emergency packet will be blocked. Emergency packets’ blocking rate $\beta_{1}$ is expressed as follows.

(19) $$\beta_{1}=\sum_{x=1}^{K+1}\pi_{x,1,1}{\bar{s}}_{1}p_{1}$$

#### 4.1.2. Emergency Packets’ Throughput

Emergency packets’ throughput $\theta_{1}$ is the amount of emergency packets forwarded completely in a unit time slot, expressed as follows.

(20) $$\theta_{1}=p_{1}-\beta_{1}$$

### 4.2. Performance Metrics for Non−Emergency Packets

When an incoming non−emergency packet voluntarily leaves the system with probability (1−f) or requests access with probability *f* while the system has no vacancies, this incoming non−emergency packet will be blocked. Non−emergency packets’ blocking rate $\beta_{2}$ is expressed as following two situations.

1. If H=K, $\beta_{2}$ is expressed as follows.
  (21) $$\begin{array}{c} \begin{array}{cc} \beta_{2} & =\sum_{x=0}^{H}\pi_{x,0,0}p_{2}\frac{x}{\alpha }+\sum_{x=1}^{K+1}\left(\pi_{x,0,1}+\pi_{x,1,1}\right)p_{2}\frac{x}{\alpha }\\ \; & +\left(\pi_{K,0,0}+\pi_{K,0,1}{\bar{s}}_{2}+\pi_{K,1,0}{\bar{s}}_{1}\right)\left(1-\frac{K}{\alpha }\right)p_{1}p_{2}\\ \; & +\left(\pi_{K+1,0,1}\left({\bar{s}}_{2}+s_{2}p_{1}\right)+\pi_{K+1,1,1}\left({\bar{s}}_{1}+s_{1}p_{1}\right)\right)\left(1-\frac{K+1}{\alpha }\right)p_{2} \end{array} \end{array}$$
2. If 0≤H≤K−1, $\beta_{2}$ is expressed as follows.
  (22) $$\begin{array}{c} \begin{array}{cc} \beta_{2} & =\sum_{x=0}^{H}\pi_{x,0,0}p_{2}\frac{x}{\alpha }+\sum_{x=1}^{K+1}\left(\pi_{x,0,1}+\pi_{x,1,1}\right)p_{2}\frac{x}{\alpha }\\ \; & +\left(\pi_{K,0,1}{\bar{s}}_{2}+\pi_{K,1,0}{\bar{s}}_{1}\right)\left(1-\frac{K}{\alpha }\right)p_{1}p_{2}\\ \; & +\left(\pi_{K+1,0,1}\left({\bar{s}}_{2}+s_{2}p_{1}\right)+\pi_{K+1,1,1}\left({\bar{s}}_{1}+s_{1}p_{1}\right)\right)\left(1-\frac{K+1}{\alpha }\right)p_{2} \end{array} \end{array}$$

When a non−emergency packet is interrupted, the interrupted packet will be discarded if there are no available spaces in the cache. Non−emergency packets’ outage and loss rate $\phi_{2}$ is expressed as follows.

(23) $$\phi_{2}=\pi_{K+1,0,1}{\bar{s}}_{2}p_{1}$$

Non−emergency packets’ throughput $\theta_{2}$ is the amount of emergency packets forwarded completely in a unit time slot, expressed as follows.

(24) $$\theta_{2}=p_{2}-\beta_{2}-\phi_{2}$$

Non−emergency packets’ average latency $\eta_{2}$ refers to the time interval between non−emergency packets entering and leaving the system. According to Little’s formula [28], $\eta_{2}$ is expressed as following two situations.

1. If H=0, $\eta_{2}$ is expressed as follows.
  (25) $$\eta_{2}=\frac{\sum_{x=1}^{K+1}\left(\pi_{x,0,1}x+\pi_{x,1,1}\left(x-1\right)\right)}{p_{2}-\beta_{2}}$$
2. If 1≤H≤K, $\eta_{2}$ is expressed as follows.
  (26) $$\eta_{2}=\frac{\sum_{x=1}^{H}\pi_{x,0,0}x+\sum_{x=1}^{K+1}\left(\pi_{x,0,1}x+\pi_{x,1,1}\left(x-1\right)\right)}{p_{2}-\beta_{2}}$$

### 4.3. Performance Metrics for State Switching and Energy Consumption

The state switching rate ϵ is the amount of times the CH state switches in a unit time slot, expressed as following two situations.

1. If H=0, ϵ is expressed as follows.
  (27) $$\epsilon =\pi_{0,0,0}\left(1-{\bar{p}}_{1}{\bar{p}}_{2}\right)+\pi_{1,0,1}s_{2}{\bar{p}}_{1}{\bar{p}}_{2}+\pi_{1,1,1}s_{1}{\bar{p}}_{1}{\bar{p}}_{2}$$
2. If 1≤H≤K, ϵ is expressed as follows.
  (28) $$\epsilon =\sum_{x=0}^{H-1}\pi_{x,0,0}p_{1}+\pi_{H,0,0}\left(1-{\bar{p}}_{1}\left({\bar{p}}_{2}+p_{2}\frac{H}{\alpha }\right)\right)+\pi_{1,0,1}s_{2}{\bar{p}}_{1}{\bar{p}}_{2}+\pi_{1,1,1}s_{1}{\bar{p}}_{1}{\bar{p}}_{2}$$

The definition of energy consumption *E* is the energy consumed by the CH per unit time slot. Considering that the state switching of the CH requires a certain amount of energy to complete, the energy consumption *E* of the CH is expressed in the following two situations.

1. If H=0, ρ is expressed as follows.
  (29) $$\begin{array}{c} \begin{array}{cc} E & =\pi_{0,0,0}{\bar{p}}_{1}{\bar{p}}_{2}E_{1}+\pi_{0,0,0}\left(1-{\bar{p}}_{1}{\bar{p}}_{2}\right)E_{2}+\left(\pi_{1,0,1}s_{2}{\bar{p}}_{1}{\bar{p}}_{2}+\pi_{1,1,1}s_{1}{\bar{p}}_{1}{\bar{p}}_{2}\right)E_{3}\\ \; & +\left(\pi_{1,0,1}\left(1-s_{2}{\bar{p}}_{1}{\bar{p}}_{2}\right)+\pi_{1,1,1}\left(1-s_{1}{\bar{p}}_{1}{\bar{p}}_{2}\right)+\sum_{x=2}^{K+1}\left(\pi_{x,0,1}+\pi_{x,1,1}\right)\right)E_{4} \end{array} \end{array}$$
2. If 1≤H≤K, ρ is expressed as follows.
  (30) $$\begin{array}{c} \begin{array}{cc} E & =\sum_{x=0}^{H-1}\pi_{x,0,0}\left({\bar{p}}_{1}+\pi_{H,0,0}{\bar{p}}_{1}\left({\bar{p}}_{2}+p_{2}\frac{H}{\alpha }\right)\right)E_{1}\\ \; & +\left(\sum_{x=0}^{H-1}\pi_{x,0,0}p_{1}+\pi_{H,0,0}\left(1-{\bar{p}}_{1}\left({\bar{p}}_{2}+p_{2}\frac{H}{\alpha }\right)\right)\right)E_{2}\\ \; & +\left(\pi_{1,0,1}s_{2}{\bar{p}}_{1}{\bar{p}}_{2}+\pi_{1,1,1}s_{1}{\bar{p}}_{1}{\bar{p}}_{2}\right)E_{3}\\ \; & +\left(\pi_{1,0,1}\left(1-s_{2}{\bar{p}}_{1}{\bar{p}}_{2}\right)+\pi_{1,1,1}\left(1-s_{1}{\bar{p}}_{1}{\bar{p}}_{2}\right)+\sum_{x=2}^{K+1}\left(\pi_{x,0,1}+\pi_{x,1,1}\right)\right)E_{4} \end{array} \end{array}$$

where $E_{1}$ is the energy consumption in a unit time slot for keeping the CH in the sleep state; $E_{2}$ is the energy consumption for changing the CH from the sleep state to working state; $E_{3}$ is the energy consumption for changing the CH from the working state to sleep state; and $E_{4}$ is the energy consumption for keeping the CH in the working state.

## 5. Numerical Results

### 5.1. Performance Analysis

To evaluate how various parameters affect the system’s performance, numerical experiments are conducted to obtain various performance index figures. In the numerical experiments, the fixed parameters are displayed in Table 3. Other dynamic parameters are displayed in the legends, while the wakeup threshold *H* is set as the horizontal axis parameter.

#### 5.1.1. Performance Analysis for Emergency Packets

**Figure 5:**
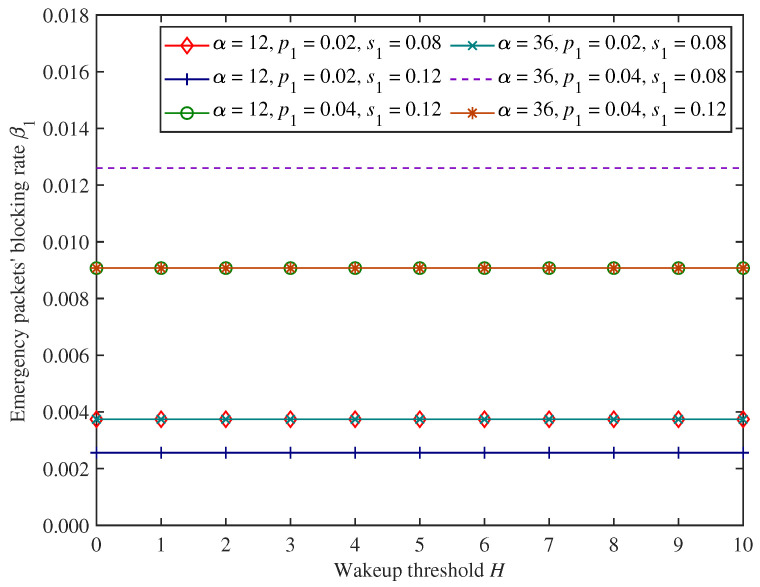
The changing trends in emergency packets’ blocking rate.

**Figure 6:**
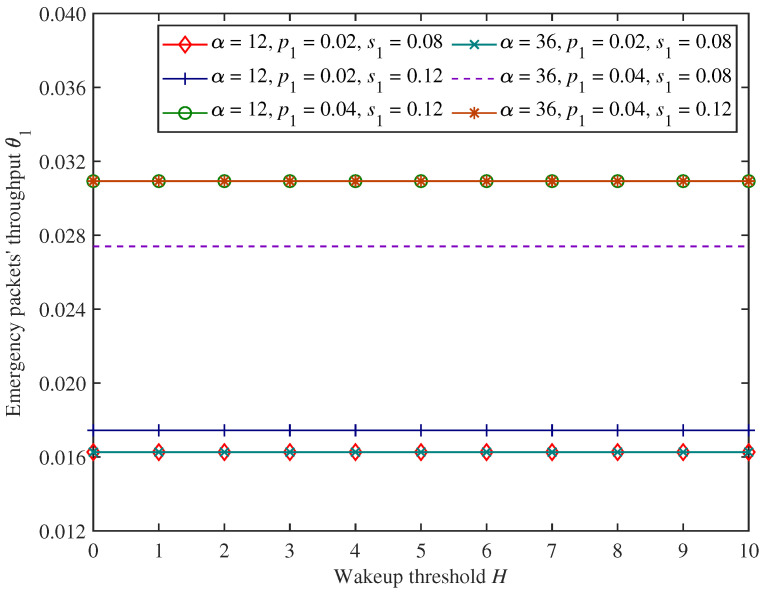
The changing trends in emergency packets’ throughput.

Figure 5 and Figure 6 show the changing trends in emergency packets’ blocking rate $\beta_{1}$ and throughput $\theta_{1}$ under the proposed mixed mechanism.

As shown in Figure 5 and Figure 6, when the wakeup threshold *H* and adjustment factor α change, emergency packets’ blocking rate and throughput remain unchanged. This is because the wakeup threshold and dynamic access probability control the access and forwarding process for only non−emergency packets, and the system behaviour of non−emergency packets cannot affect emergency packets, which have preemptive priority. It also indicates that the proposed mixed mechanism can ensure the comprehensive forwarding performance of emergency packets.

In Figure 5 and Figure 6, we can find that as emergency packets’ arrival rate $p_{1}$ increases, emergency packets’ blocking rate and throughput also increase under the parameter settings of this experiment. This is because the CH can forward a greater number of emergency packets if the arrival rate of emergency packets is higher, and emergency packets’ throughput will be larger. However, the forwarding efficiency of the CH is constant, and there will also be more incoming emergency packets that cannot access the CH.

Moreover, in Figure 5 and Figure 6, when emergency packets’ service rate $s_{1}$ increases, emergency packets’ blocking rate decreases, and the throughput increases under the parameter settings of this experiment. This is because the higher the emergency packets’ service rate, the more emergency packets can be forwarded completely by the CH per unit of time, and the corresponding number of non−emergency packets that the CH can forward increases, so emergency packets’ throughput will increase. This also provides more opportunities for emergency packets to access CH for data forwarding and reduces their blocking rate.

#### 5.1.2. Performance Analysis for Non−Emergency Packets

**Figure 7:**
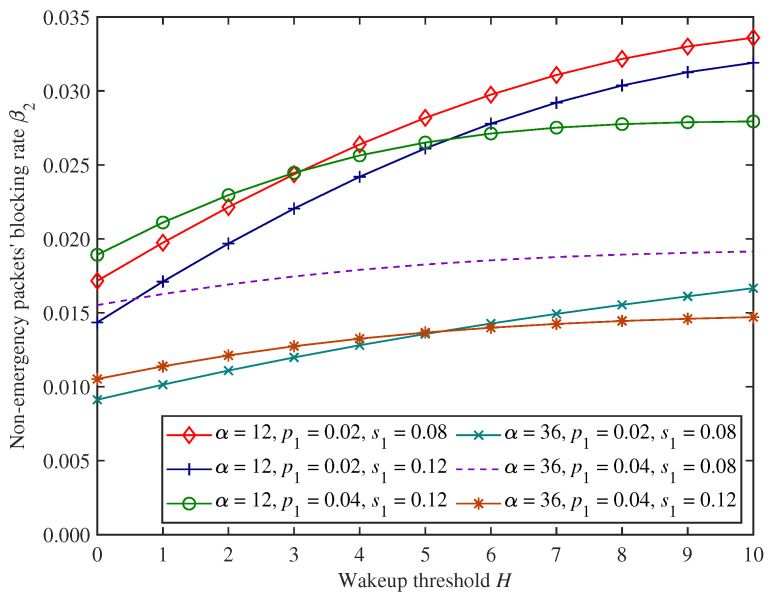
The changing trends in non−emergency packets’ blocking rate.

**Figure 8:**
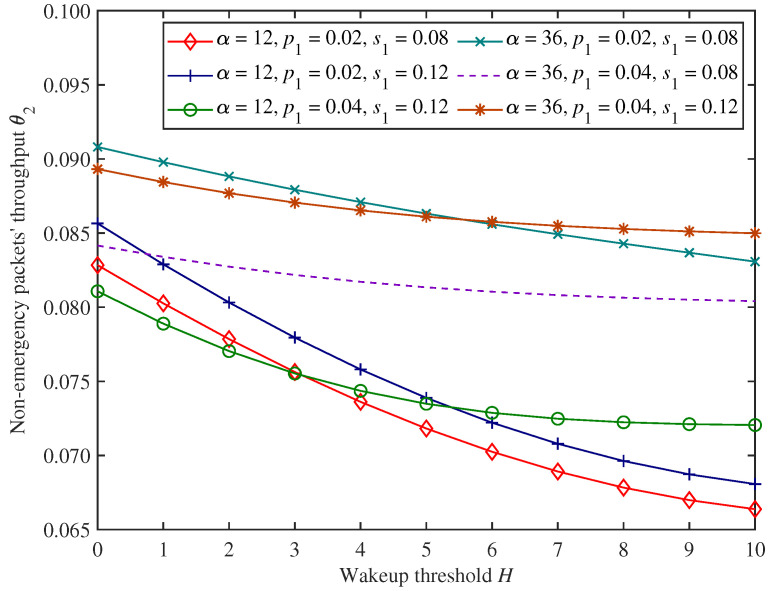
The changing trends in non−emergency packets’ throughput.

**Figure 9:**
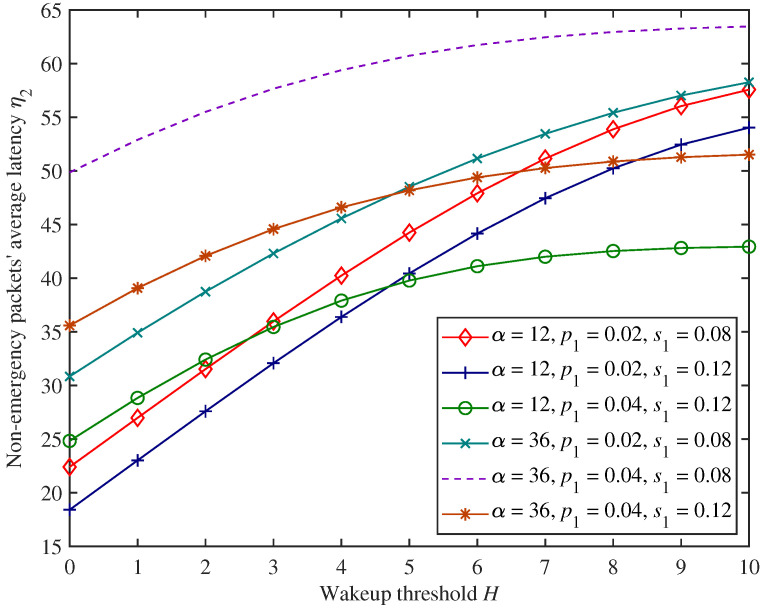
The changing trends in non−emergency packets’ average latency.

Figure 7, Figure 8 and Figure 9 show the change trends in non−emergency packets’ blocking rate $\beta_{2}$, throughput $\theta_{2}$, and average latency $\eta_{2}$ under the proposed mixed mechanism.

In Figure 7, Figure 8 and Figure 9, as the wakeup threshold *H* increases, non−emergency packets’ blocking rate and average latency show an upward trend. In contrast, the throughput shows a downward trend under the parameter settings of this experiment. This is because as the wakeup threshold increases, the CH will start forwarding non−emergency packets only when the amount of non−emergency packets stuck in the system increases. The number of successfully forwarded non−emergency packets per unit time decreases, resulting in a decrease in throughput. At the same time, more non−emergency packets are stuck in the cache, and the possibility of the cache being full is also higher, so the blocking rate and average latency both increase.

Moreover, in Figure 7, Figure 8 and Figure 9, when the adjustment factor α increases, non−emergency packets’ blocking rate decreases, while throughput and average latency increase under the parameter settings of this experiment. This has to do with the fact that dynamic access probability *f* is the probability of allowing non−emergency packets to access the system dynamically based on the load, and the larger the adjustment factor α, the higher the dynamic access probability. Therefore, the amount of non−emergency packets that voluntarily abandon access to the system for forwarding decreases, resulting in a corresponding decrease in blocking rate and an increase in throughput. At the same time, a large number of non−emergency packets are stuck in the cache after accessing the system, increasing average latency.

In addition, from Figure 7, Figure 8 and Figure 9, when emergency packets’ arrival rate $p_{1}$ increases, non−emergency packets’ blocking rate and average latency also increase under the parameter settings of this experiment and throughput decreases. This is because, in this situation, the CH is more likely to be occupied by emergency packets, while a large number of non−emergency packets are stuck in the cache waiting to be forwarded, resulting in an increase in blocking rate and average latency and a decrease in throughput. Similarly, emergency packets’ service rate has an opposite impact on the forwarding performance of non−emergency packets compared with the arrival rate.

#### 5.1.3. Performance Analysis for State Switching and Energy Consumption

**Figure 10:**
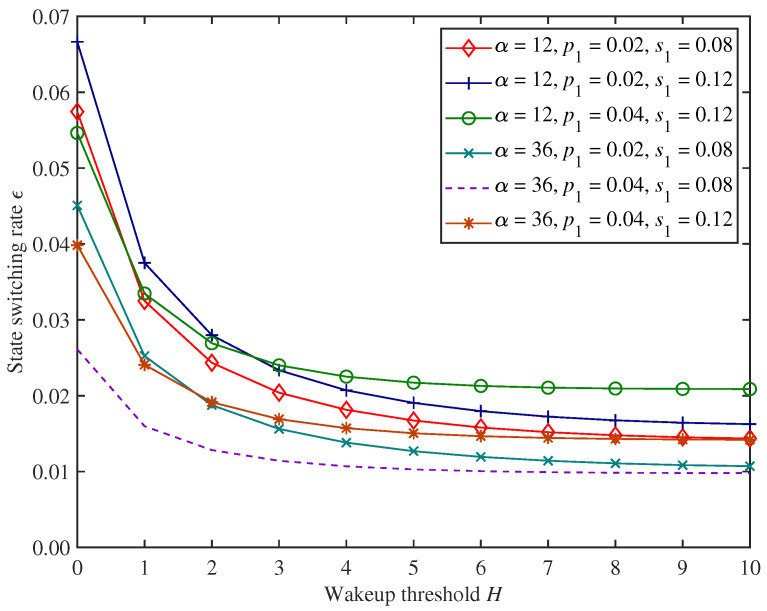
The changing trends in state switching rate of the CH.

**Figure 11:**
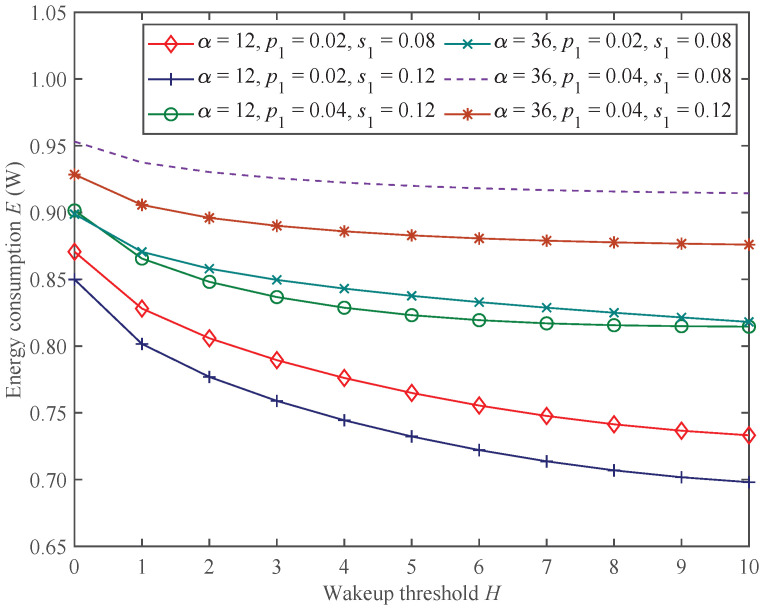
The changing trends in energy consumption of the CH.

Figure 10 and Figure 11 show the change trends in the state switching rate ϵ and energy consumption *E* under the proposed mixed mechanism.

In Figure 10 and Figure 11, we can find that as the wakeup threshold *H* increases, the state switching rate ϵ and energy consumption *E* of the CH show decreasing trends under the parameter settings of this experiment. This is because the wakeup threshold constrains the conditions for the CH to switch from sleep state to working state. The larger the wakeup threshold, the more time the CH will remain in the sleep state, resulting in a lower state switching rate and energy consumption.

Moreover, in Figure 10 and Figure 11, under the parameter settings of this experiment, increasing the adjustment factor α will reduce the state switching rate while increasing energy consumption instead. According to the definition of access probability, the larger the adjustment factor, the greater the possibility of allowing non−emergency packets to access the CH. At this time, the CH remains in the working state for a longer period of time, reducing the possibility of switching to the sleep state, thereby reducing the state switching rate and increasing energy consumption. Similarly, increasing emergency packets’ arrival rate $p_{1}$ or decreasing service rate $s_{1}$ shows the same trends on state switching rate and energy consumption.

### 5.2. Performance Comparison

To evaluate the effectiveness of the proposed mixed mechanism, a performance comparison experiment is conducted between the proposed mixed mechanism and existing mechanisms using numerical results. We compare non−emergency packets’ average latency, state switching rate, and energy consumption with respect to whether to use the wakeup threshold mechanism [19] or the dynamic access probability mechanism (which can be regarded as a variation of the RED [23] method where the minimum threshold is 0 and maximum threshold is equal to the cache size *K*). Due to the adjustability of the proposed mixed mechanism, the existing mechanisms for comparison can be obtained by changing the values of the adjustment factor α and wakeup threshold *H*. The differences between contrast mechanisms are shown in Table 4. The parameter settings of different mechanisms are shown in Table 5, and other fixed parameters are shown in Table 6.

**Figure 12:**
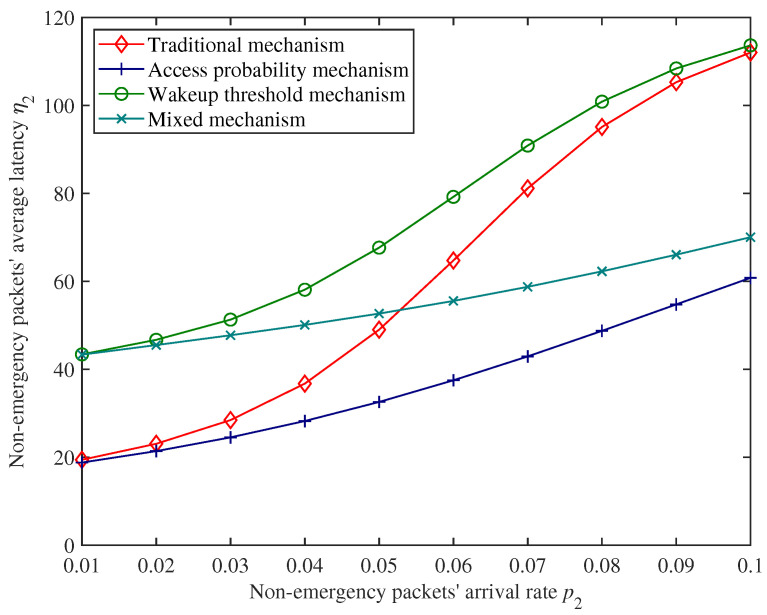
Comparison for non−emergency packets’ average latency.

**Figure 13:**
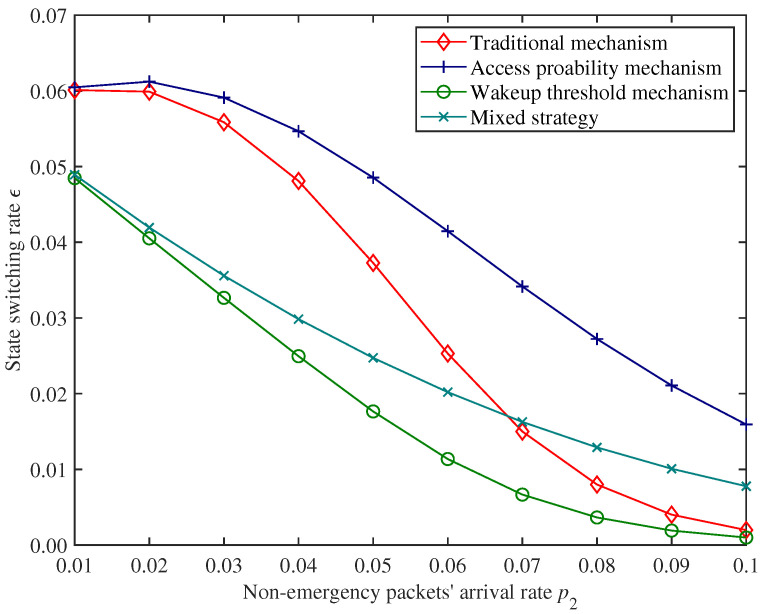
Comparison for switching rate of the CH.

**Figure 14:**
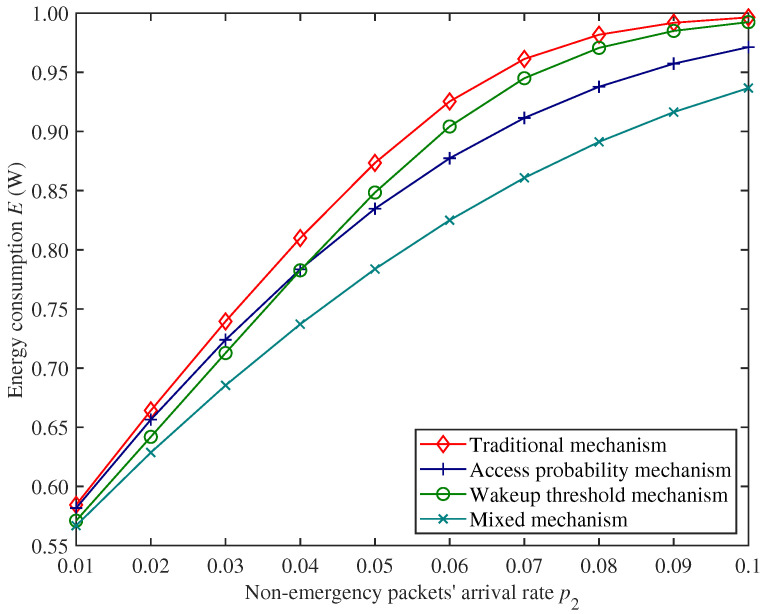
Comparison energy consumption of the CH.

Figure 12, Figure 13 and Figure 14 show the performance comparison of non−emergency packets’ average latency $\eta_{2}$, state switching rate ϵ, and energy consumption *E* of the CH under different mechanisms.

From Figure 12 and Figure 13, compared with the traditional mechanism, introducing an access probability mechanism can effectively reduce the non−emergency packets’ average latency, but this will also increase the state switching rate of the CH accordingly. On the contrary, introducing a wakeup threshold mechanism can reduce the state switching rate of the CH but also increase non−emergency packets’ average latency. Therefore, introducing a single mechanism cannot effectively balance these two performance indexes.

Moreover, in Figure 12, Figure 13 and Figure 14, under the parameter settings of this experiment, the performance of the traditional mechanism of the CH shows a polarized trend when the non−emergency packets arrival rate is at a low level and high level. With the increase in non−emergency packets’ arrival rate, the state switching rate of the CH shows a drastic change. The mixed mechanism proposed in this paper has relatively smooth trends for these two performance indexes and is more stable under different packet loads. (We use range to express the difference between the maximum value and the minimum value. The range of $\eta_{2}$ under the traditional mechanism is approximately 92.630, while the range of $\eta_{2}$ under the proposed mechanism is approximately 26.706; the range of ϵ under traditional mechanism is approximately 0.058, while the range of ϵ under the proposed mechanism is approximately 0.041). In addition, within a certain range (for example, when $0.053<p_{2}<0.068$), non−emergency packets’ average latency and state switching rate of the CH under the mixed mechanism are both lower than those under the traditional mechanism.

In Figure 14, we can see that both the access probability mechanism and the wakeup threshold mechanism can save system energy, and the energy consumption of the proposed mechanism, which introduces both mechanisms, is the lowest. This also demonstrates the effectiveness of the mixed mechanism proposed in this paper for energy conservation.

In summary, compared to using only the access probability mechanism or wakeup threshold mechanism, the mixed mechanism proposed in this paper can effectively balance non−emergency packets’ latency and state switching rate of the CH. Compared with the traditional mechanism, the mixed mechanism proposed in this paper has stronger stability in performance under different packet loads. Within a certain load range, the proposed mixed mechanism outperforms traditional mechanisms in both latency and state switching rate.

## 6. Conclusions

To effectively balance the latency and energy consumption performance while meeting the diverse transmission needs of data in UWSNs, we proposed a mixed packet forwarding strategy based on wakeup threshold and dynamic access probability with priority scheduling for CHs. We derived a series of system performance index expressions by constructing a discrete−time queueing model with preemptive priority. We provided numerical results and analyzed how various parameters affected network performance. By comparing with the traditional mechanism, access probability mechanism, and wakeup threshold mechanism, we objectively analyzed the superiority of the proposed mixed mechanism. The numerical results indicated that compared with the traditional mechanism, the mixed mechanism proposed in this paper was less affected by network load in terms of latency and energy consumption (for example, the range of $\eta_{2}$ under traditional mechanism is approximately 92.630, while the range of $\eta_{2}$ under the proposed mechanism is approximately 26.706; the range of ϵ under traditional mechanism is approximately 0.058, while the range of ϵ under the proposed mechanism is approximately 0.041), and performs better than traditional mechanisms within a specific parameter range (for example, when $0.053<p_{2}<0.068$). Compared with the single access probability mechanism and wakeup threshold mechanism, the mixed mechanism could effectively balance latency and energy consumption performance.

In this paper, we assume that the arrival process of packets is a single−input process that obeys the geometric distribution, and in future work, we can further construct a parallel−input queueing model to analyze the network performance with multiple sensor nodes, so as to improve the accuracy of the model. In addition, in this paper, our numerical results are derived from the ideal environment. In future work, we will consider non−ideal conditions such as forwarding failures or mistakes, and further build a queueing model that is more in line with the actual network environment. Moreover, this paper only divides the CH into two states (sleep/working mode). In future research, we will consider further dividing it into multi−state mode (such as sleep/idle/working mode [30]) according to the actual environment to improve the accuracy of our model.

## Figures and Tables

**Table 1:** Symbols and corresponding meanings.

Symbols	Means
*K*	Cache capacity
*f*	Access probability
*N*	Amount of packets in the system
$f_{min}$	Minimum value of *f*
α	Adjustment factor for the *f*
*H*	Wakeup threshold
*t*	Index of time slots
$p_{1}$	Emergency packets’ arrival rate at the CH
$p_{2}$	Non−emergency packets’ arrival rate at the CH
$s_{1}$	Emergency packets’ service rate at the CH
$s_{2}$	Non−emergency packets’ service rate at the CH
$R_{t}$	Amount of total packets at time $t^{+}$
$S_{t}$	Amount of emergency packets at time $t^{+}$
$T_{t}$	CH’s state at time $t^{+}$
*i*, *j*, *k*	Three indexes of the system state
Ω	State space
W	One−step transition probability matrix
*m*	Amount of total packets before one−step transition
*n*	Amount of total packets after one−step transition
$W_{m,n}$	Block matrix where the total number of packets changes from *m* to *n* in one−step transition
*x*, *y*, *z*	Indexes of the steady−state probability distribution
$\pi_{x,y,z}$	Steady−state probability distribution
Π	Steady−state vector
e, $e^{\prime }$	Two intermediate vectors for solving $\pi_{x,y,z}$
Q, $Q^{\prime }$	Two intermediate matrixes for solving $\pi_{x,y,z}$
$\beta_{1}$	Emergency packets’ blocking rate
$\theta_{1}$	Emergency packets’ throughput
$\beta_{2}$	Non−emergency packets’ blocking rate
$\phi_{2}$	Non−emergency packets’ outage and loss rate
$\theta_{2}$	Non−emergency packets’ throughput
$\eta_{2}$	Non−emergency packets’ average latency
ϵ	State switching rate
*E*	Energy consumption
$E_{1}$, $E_{2}$, $E_{3}$, $E_{4}$	Energy consumption in different states

**Table 2:** The procedures for solving $\pi_{x,y,z}$.

Step 1	Create unit matrix $Q={\left(\begin{array}{ccc} 1 & & \\ & \ddots & \\ & & 1 \end{array}\right)}_{\left(2K+H+3)\times (2K+H+3\right)}$.
Step 2	Create column vector $e={\left(\begin{array}{c} 1\\ \vdots \\ 1 \end{array}\right)}_{(2K+H+3)\times 1}$.
Step 3	Create matrix $Q^{\prime }=\left(W-Q,e\right)$.
Step 4	Create row vector $e^{\prime }={\left(0,\cdots ,0,1\right)}_{1\times (2K+H+4)}$, which contains (2K+H+3) zeros.
Step 5	Resolve $\Pi =e^{\prime }/Q^{\prime }$.

**Table 3:** The fixed parameters of the experiments for performance analysis.

Fixed Parameters	Symbols	Values
Cache capacity	*K*	10
Non−emergency packets arrival rate at CH	$p_{2}$	0.10
Non−emergency packets’ service rate at CH	$s_{2}$	0.15
Energy consumption for keeping CH in sleep state	$E_{1}$	0.20 (W)
Energy consumption for changing CH from sleep state to working state	$E_{2}$	2 (W)
Energy consumption for changing CH from working state to sleep state	$E_{3}$	1.50 (W)
Energy consumption for keeping CH in working state	$E_{4}$	1 (W)

**Table 4:** Differences between contrast mechanisms.

Mechanism Names	Tail−Drop	Sleep/ Wakeup Mode	Wakeup Threshold	Dynamic Access Probability
Traditional mechanism	Yes	Yes	No	No
Dynamic access probability mechanism	Yes	Yes	No	Yes
Wakeup threshold mechanism	Yes	Yes	Yes	No
Mixed mechanism	Yes	Yes	Yes	Yes

**Table 5:** The values of adjustment factor and wakeup threshold under different mechanisms.

Mechanism Names	Wakeup Threshold *H*	Adjustment Factor α	Access Probability *f*
Traditional mechanism	0	*∞*	1
Dynamic access probability mechanism	0	12	auto
Wakeup threshold mechanism	5	*∞*	1
Mixed mechanism	5	12	auto

**Table 6:** The fixed parameters of the experiment for performance comparison.

Fixed Parameters	Symbols	Values
ine Cache capacity	*K*	10
Emergency packets’ arrival rate	$p_{1}$	0.04
Emergency packets’ service rate at CH	$s_{1}$	0.10
Non−emergency packets’ service rate at CH	$s_{2}$	0.15
Energy consumption for keeping CH in sleep state	$E_{1}$	0.20 (W)
Energy consumption for changing CH from sleep state to working state	$E_{2}$	2 (W)
Energy consumption for changing CH from working state to sleep state	$E_{3}$	1.50 (W)
Energy consumption for keeping CH in working state	$E_{4}$	1 (W)

## Data Availability

All relevant data are within the paper.

## References

[1] Khan M.U., Otero P., Aamir M. Underwater Acoustic Sensor Networks (UASN): Energy Efficiency Perspective of Cluster-Based Routing Protocols. Proceedings of the 2022 Global Conference on Wireless and Optical Technologies (GCWOT).

[2] Tian W., Zhao Y., Hou R., Dong M., Ota K., Zeng D., Zhang J. (2023). A centralized control-based clustering scheme for energy efficiency in underwater acoustic sensor networks. IEEE Trans. Green Commun. Netw..

[3] Khalid M., Ullah Z., Ahmad N., Arshad M., Jan B., Cao Y., Adnan A. (2017). A survey of routing issues and associated protocols in underwater wireless sensor networks. J. Sens..

[4] Luo J., Chen Y., Wu M., Yang Y. (2021). A survey of routing protocols for underwater wireless sensor networks. IEEE Commun. Surv. Tutorials.

[5] Murgod T.R., Sundaram S.M., Manchaiah S., Kumar S. (2023). Priority based energy efficient hybrid cluster routing protocol for underwater wireless sensor network. Int. J. Electr. Comput. Eng. (IJECE).

[6] Sun Y., Zheng M., Han X., Li S., Yin J. (2022). Adaptive clustering routing protocol for underwater sensor networks. Ad Hoc Netw..

[7] Liu G., Yan S., Mao L. (2020). Receiver-Only-Based Time Synchronization Under Exponential Delays in Underwater Wireless Sensor Networks. IEEE Internet Things J..

[8] Gomathi R., Manickam J.M.L., Sivasangari A., Ajitha P. (2020). Energy efficient dynamic clustering routing protocol in underwater wireless sensor networks. Int. J. Netw. Virtual Organ..

[9] Subramani N., Mohan P., Alotaibi Y., Alghamdi S., Khalaf O.I. (2022). An efficient metaheuristic-based clustering with routing protocol for underwater wireless sensor networks. Sensors.

[10] Raina V., Jha M.K., Bhattacharya P.P. (2017). The Alive-in-Range Medium Access Control Protocol to Optimize Queue Performance in Underwater Wireless Sensor Networks. J. Telecommun. Inf. Technol..

[11] Domingo M.C. (2013). Marine communities based congestion control in underwater wireless sensor networks. Inf. Sci..

[12] Goyal N., Dave M., Verma A.K. (2016). Congestion control and load balancing for cluster based underwater wireless sensor networks. Proceedings of the 2016 Fourth International Conference on Parallel, Distributed and Grid Computing (PDGC).

[13] Luo Y., Dong Y., Zhu X., Chen Y., Wu J. (2023). AUV-Assisted Data Collection Based on Queuing Theory and Genetic Algorithm for Underwater Acoustic Cooperative Sensor Networks. Proceedings of the 2023 IEEE International Conference on Signal Processing, Communications and Computing (ICSPCC).

[14] Al-Halafi A., Alghadhban A., Shihada B. (2019). Queuing Delay Model for Video Transmission Over Multi-Channel Underwater Wireless Optical Networks. IEEE Access.

[15] Lin C., Wang K., Chu Z., Wang K., Deng J., Obaidat M.S., Wu G. (2018). Hybrid charging scheduling schemes for three-dimensional underwater wireless rechargeable sensor networks. J. Syst. Softw..

[16] Alfa A.S. (2010). Queueing Theory for Telecommunications: Discrete Time Modelling of a Single Node System.

[17] Ye D., Zhang M. (2018). A Self-Adaptive Sleep/Wake-Up Scheduling Approach for Wireless Sensor Networks. IEEE Trans. Cybern..

[18] Poostpasand M., Javidan R. (2018). An adaptive target tracking method for 3D underwater wireless sensor networks. Wirel. Netw..

[19] Zhang C., Yang J., Wang N. (2024). An active queue management for wireless sensor networks with priority scheduling strategy. J. Parallel Distrib. Comput..

[20] Abualhaj M.M., Abu-Shareha A.A., Al-Tahrawi M.M. (2018). FLRED: An efficient fuzzy logic based network congestion control method. Neural Comput. Appl..

[21] Karmeshu, Patel S., Bhatnagar S. (2017). Adaptive mean queue size and its rate of change: Queue management with random dropping. Telecommun. Syst..

[22] Feng C.W., Huang L.F., Xu C., Chang Y.C. (2015). Congestion control scheme performance analysis based on nonlinear RED. IEEE Syst. J..

[23] Xu Y., Qi H., Xu T., Hua Q., Yin H., Hua G. (2019). Queue models for wireless sensor networks based on random early detection. Peer-Netw. Appl..

[24] Zhang X., Li D., Zhang Y. (2020). Maximum throughput under admission control with unknown queue-length in wireless sensor networks. IEEE Sens. J..

[25] Huang D.C., Lee J.H. (2013). A dynamic N threshold prolong lifetime method for wireless sensor nodes. Math. Comput. Model..

[26] Keshtgary M., Mohammadi R., Mahmoudi M., Mansouri M.R. (2012). Energy consumption estimation in cluster based underwater wireless sensor networks using m/m/1 queuing model. Int. J. Comput. Appl..

[27] Zhao Y., Li H., Liu J. (2019). Performance analysis and optimization of CRNs based on fixed feedback probability mechanism with two classes of secondary users. Math. Probl. Eng..

[28] Tian N., Zhang Z.G. (2006). Vacation Queueing Models: Theory and Applications.

[29] Zhao Y., Xiang Z., Lu Q. (2022). Performance evaluation for secondary users in finite-source cognitive radio networks with dynamic preemption limit. AEU-Int. J. Electron. Commun..

[30] You S., Eshraghian J.K., Iu H.C., Cho K. (2021). Low-power wireless sensor network using fine-grain control of sensor module power mode. Sensors.

